# Pirfenidone in patients with rapidly progressive interstitial lung disease associated with clinically amyopathic dermatomyositis

**DOI:** 10.1038/srep33226

**Published:** 2016-09-12

**Authors:** Ting Li, Li Guo, Zhiwei Chen, Liyang Gu, Fangfang Sun, Xiaoming Tan, Sheng Chen, Xiaodong Wang, Shuang Ye

**Affiliations:** 1 Department of Rheumatology, Ren Ji Hospital South Campus, School of Medicine, Shanghai JiaoTong University, Shanghai, 200001, China; 2 Department of Pulmonology, Ren Ji Hospital South Campus, School of Medicine, Shanghai JiaoTong University, Shanghai, 200001, China

## Abstract

To evaluate the efficacy of pirfenidone in patients with rapidly progressive interstitial lung disease (RPILD) related to clinically amyopathic dermatomyositis (CADM), we conducted an open-label, prospective study with matched retrospective controls. Thirty patients diagnosed with CADM-RPILD with a disease duration <6 months at Renji Hospital South Campus from June 2014 to November 2015 were prospectively enrolled and treated with pirfenidone at a target dose of 1800 mg/d in addition to conventional treatment, such as a glucocorticoid and/or other immunosuppressants. Matched patients without pirfenidone treatment (n = 27) were retrospectively selected as controls between October 2012 and September 2015. We found that the pirfenidone add-on group displayed a trend of lower mortality compared with the control group (36.7% vs 51.9%, *p* = 0.2226). Furthermore, the subgroup analysis indicated that the pirfenidone add-on had no impact on the survival of acute ILD patients (disease duration <3 months) (50% vs 50%, *p* = 0.3862); while for subacute ILD patients (disease duration 3–6 months), the pirfenidone add-on (n = 10) had a significantly higher survival rate compared with the control subgroup (n = 9) (90% vs 44.4%, *p* = 0.0450). Our data indicated that the pirfenidone add-on may improve the prognosis of patients with subacute ILD related to CADM.

Interstitial lung disease (ILD) is a common complication of dermatomyositis (DM) with a prevalence rate as great as 65% and is considered a key prognostic factor^1^. Clinical amyopathic dermatomyositis (CADM), which is a special phenotype of DM with characteristic cutaneous manifestations but no or only subclinical myopathy^2^. Many studies, mainly from Asia, including ours, have demonstrated that patients with CADM tend to develop rapidly progressive ILD (RPILD) and have a poor response to conventional therapy, such as high-dose glucocorticoids and immunosuppressants, leading to lethal outcomes with a 6-month survival rate of less than 50%^3^,^4^,^5^.

Pirfenidone, a new oral antifibrotic agent, has been approved for the treatment of idiopathic pulmonary fibrosis (IPF). Randomized controlled trials of pirfenidone in patients with IPF suggested that it could ameliorate pulmonary function decline and improve progression-free survival^6^,^7^,^8^. Its utility in ILD related to connective tissue disease (CTD) has been implicated^9^, but no evidence has yet demonstrated its efficacy. Therefore, we conducted this study to evaluate the possible therapeutic effects of pirfenidone on RPILD associated with CADM.

## Results

The baseline clinical features of the pirfenidone add-on group (n = 30) and the matched control group (n = 27) are summarized in Table 1.

### Outcomes

**Figure 1:**
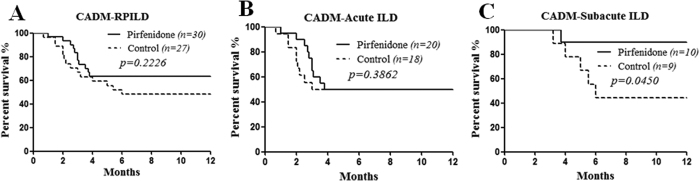
The survival curves of the pirfenidone add-on group and the control group. (**A**) Survival analysis of all patients showed that the pirfenidone add-on group had fewer deaths than the control group, but the difference was not significant. (**B**) The pirfenidone add-on had no impact on the survival of patients with acute ILD (disease duration <3 months). (**C**) The pirfenidone add-on led to a significantly higher survival rate in subacute ILD patients (disease duration 3–6 months) compared with the control subgroup.

**Figure 2:**
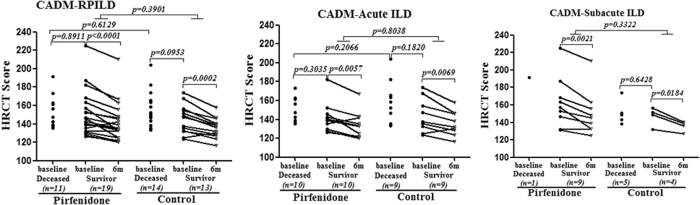
The overall HRCT score at baseline and its changes at 6 months among survivors. The baseline level did not significantly differ between the pirfenidone and control groups or the deceased and survivor groups.

The analysis of all-cause mortality in all patients showed fewer deaths in the pirfenidone add-on group than in the control group, although the difference was not significant (36.7% vs 51.9%, *p* = 0.2226) (Fig. 1A). After dividing the patients into two subgroups according to the duration of ILD, the pirfenidone add-on had no impact on the survival of patients with acute ILD (disease duration <3 months) (50% vs 50%, *p* = 0.3862) (Fig. 1B); while in patients with subacute ILD (disease duration between 3–6 months), the pirfenidone add-on (n = 10) achieved a significantly higher survival rate compared with the control subgroup (n = 9) (90% vs 44.4%, *p* = 0.0450) (Fig. 1C). The baseline HRCT scores were not different between the groups, which further suggested the matching quality of the controls (Fig. 2). However, no additional gain in the HRCT score improvement was observed among the survivors who received pirfenidone compared to the control survivors. Changes in the %FVC were not analyzed because up to 36.8% of the baseline data were unavailable owing to the severity of the respiratory failure of these patients. Among 26 serum samples available from pirfenidone add-on group, 84.6% displayed a positive anti-melanoma differentiation-associated gene 5 autoantibody (anti-MDA-5 Ab) reaction. In retrospective control group, only limited serum samples were available (n = 7) with a positivity of anti-MDA-5 Ab at 57.1% (*p* = 0.145). Moreover, in pirfenidone add-on group, the positivity of anti-MDA-5 Ab had no significant difference between survivor and deceased (80.0% vs 90.9%, *p* = 0.617).

### Safety

No direct pirfenidone-related serious adverse events occurred during follow-up. The elevations of hepatic enzyme (30%) and gastrointestinal reaction (13.3%) were common, but most of these events were mild to moderate in severity and were reversible. Three adverse events (10%) led to treatment discontinuation in 3 survivors (2 patients with acute ILD and 1 patient with subacute ILD) and were elevated hepatic enzyme levels, rash and diarrhea.

## Discussion

According to our previous retrospective cohort study^5^, the rate of ILD in DM is as high as 50%. As a subtype of DM, CADM patients appear to be at a higher risk of developing rapidly progressive ILD, especially in the presence of the anti-MDA-5 antibody^10^,^11^. Most of the patients with CADM-RPILD are resistant to intensive therapy, such as high-dose glucocorticoids and immunosuppressive agents, resulting in over 50% mortality rate^2^,^3^,^4^,^5^. Cyclosporin alone or combined with cyclophosphamide, tacrolimus, rituximab, basiliximab, or polymyxin B hemoperfusion has been used in case reports or small case series with undetermined efficacy^12^,^13^,^14^,^15^,^16^,^17^.

To the best of our knowledge, this is the first report to investigate the efficacy of pirfenidone on CADM-RPILD patients. Among the pirfenidone add-on group, 84.6% were anti-MDA-5 autoantibody positive, which is the most representative poor prognostic marker. It has been well conceived that anti-MDA-5 antibody is strongly associated with the development of rapidly progressive, fatal ILD in CADM patients^10^,^11^,^18^. Our data indicated that the pirfenidone add-on probably has no impact on the survival of such high risk patients with acute ILD related to CADM; however, for patients with subacute ILD (disease duration between 3–6 months), pirfenidone may help suppress the disease progression and, ultimately, improve the outcome. It is noteworthy that it will take weeks and up to 3 months for pirfenidone to exert its therapeutic effects in chronic IPF. Its “slow-acting” antifibrotic property could be insufficient in the context of acute phases of lung injury, which is in line with the observation that pirfenidone is incapable of decreasing the risk of acute IPF exacerbation^19^. Moreover, according to our data, anti-MDA-5 autoantibody is apparently not an indicator of pirfenidone’s response as positivity for the antibody did not significantly differ between the survivor and deceased patients treated with pirfenidone (80.0% vs 90.9%, *p* = 0.617). In other words, it seems that only the slope of ILD progression and the phase of pathophysiology that matters. Future treatments should be developed, based on a staged approach and a timing strategy, in order to significantly change the course of this high risk disease.

The results of our study should be interpreted cautiously owing to certain limitations. First, this study was a single-center, open-label trial, and the control patients were not prospectively randomized. Second, the sample size was limited, especially for the subgroup analysis. Therefore, a multi-centered, randomized, control trial on pirfenidone targeting patients with subacute ILD secondary to CADM is needed.

## Patients and Methods

### Study design

This was a mono-centered, open-label, prospective study with matched retrospective controls. All patients fulfilled the provisional diagnosis of CADM according to the modified Sontheimer’s criteria^2^ and our previous definitions^5^. RPILD is defined as disease exacerbation within 6 months of the onset of ILD, presenting as increased levels of dyspnea and worsening of fibrosis on pulmonary HRCT with >10% increase of the HRCT score and/or a reduction in %FVC by >10% of the absolute value. Patients previously treated with biologics, including basiliximab, and patients with malignancy-associated CADM or other connective tissue disease comorbidities or with alanine transaminase levels greater than 2 times the upper normal limits were excluded.

After providing written informed consent, 30 adult patients who met the criteria between June 2014 and November 2015 at the Shanghai Renji Hospital South Campus received treatment with pirfenidone in addition to the institutional standard of care, which includes a glucocorticoid and/or immunosuppressant. Pirfenidone was administered in three divided doses (200 mg tid) and was increased to the manufacturer’s instructed target dose (600 mg tid) over a 2-week period. The maximum dose was maintained throughout the study in patients who tolerated it. Pulmonary function tests (except for cases in which the patient’s respiratory failure was too severe to perform the measurement) were performed at the start of treatment and every month thereafter. All patients underwent HRCT to assess the progression of ILD before the start of the treatment and then underwent HRCT every three months (unless there was acute exacerbation, which was determined by the investigator). The overall HRCT score was calculated based on the classification by Ichikado^20^. Matched patients meeting the same aforementioned criteria received only the institutional standard of care between October 2012 and September 2015 and were retrospectively enrolled as the control group^21^. All possible confounders, including age, gender, disease duration, the HRCT score (as a surrogate of disease severity), smoking history, exposure to a glucocorticoid or other immunosuppressant, or the use of antimicrobial/antifungal agents, were balanced using the covariance matrix of a Cox regression model. To avoid selection bias, the investigators in charge of the matched-control selection were blinded to the outcomes of each patient.

Serum anti-MDA-5 autoantibody was measured by an immune blot assay (EuroImmune, Lubeck, Germany). Briefly, each MDA5-transferred nitrocellulose strip was incubated in sample buffer on a shaker for 5 minutes at room temperature (RT) and then was incubated with the human serum sample diluted in blocking buffer for 30 minutes at RT. After washing 3 times, phosphatase alkaline-labeled goat anti-human IgG antibody was added to each strip and incubated for 30 minutes at RT. Then the strips were washed 3 times, and the phosphatase reagent, used to develop the color, was added for 10 minutes at RT before another wash. Based on the signal intensity, the results were classified as negative or positive. Krebs von den lungen-6 (KL-6), as a lung fibrosis marker, was measured by the chemiluminescent enzyme immunoassay LUMIPULSE KL-6 using a KL-6 antibody (EIDIA Co., Ltd, JPN) according to the manufacturer’s protocol.

The primary endpoint of the investigation was the changes in the 12-month survival rate from the onset of ILD. This study was conducted in accordance with the principles expressed in the Declaration of Helsinki. The study was approved by the Institutional Review Board of Renji Hospital and is registered at Chictr.org (number: ChiCTR-IPR-16007958; date: Oct 21, 2015 (retrospective registration)).

### Statistical analysis

The clinical data are expressed as the mean ± SD. Comparisons of the two groups were performed with the Mann-Whitney *U* test, the paired *t*-test, or Fisher’s exact test when indicated. The intent-to-treat analysis was performed in the survival analysis using the Kaplan-Meier test. All statistical analyses were conducted in the SPSS 19.0 software package. P < 0.05 was considered statistically significant.

## Additional Information

**How to cite this article**: Li, T. *et al*. Pirfenidone in patients with rapidly progressive interstitial lung disease associated with clinically amyopathic dermatomyositis. *Sci. Rep.* **6**, 33226; doi: 10.1038/srep33226 (2016).

## Figures and Tables

**Table 1:** Baseline characteristics of the CADM-RPIL.

Variables	Pirfenidone add-on (n = 30)	Control (n = 27)	*P*-value
Age at diagnosis (years)	46.3 ± 11.3	51.8 ± 7.8	0.124
Gender (F:M)	20: 10	15: 12	0.426
History of smoking, n (%)	9 (30.0%)	10 (37.0%)	0.589
Duration of CADM (months)	3.2 ± 2.2	2.9 ± 1.8	0.843
Duration of ILD (months)	2.3 ± 1.1	2.3 ± 1.4	0.885
Acute ILD: (<3 months) (n)	1.7 ± 0.6 (20)	1.5 ± 0.6 (18)	0.329
Subacute ILD: (3–6 months) (n)	3.7 ± 0.7 (10)	3.9 ± 1.1 (9)	0.673
ANA antibody (≥1:80), n (%)	5 (16.7%)	6 (22.2%)	0.740
Ferritin >1500 ng/ml, n (%)	11 (36.7%)	9 (33.3%)	1.000
KL-6 (U/ml)§	1238.5 ± 782.1	892.8 ± 296.0	0.627
MDA-5 antibody, n (%)§	22 (84.6%)	4 (57.1%)	0.145
CK (U/L)	91.4 ± 83.0	124.0 ± 95.6	0.244
HRCT score (%)	152.4 ± 22.4	154.2 ± 24.7	0.896
High-dose prednisolone			
>5 mg/kg, n (%)	4 (13.3%)	4 (14.8%)	1.000
1–5 mg/kg, n (%)	17 (56.7%)	14 (51.9%)	0.793
Immunosuppressant			
CsA n (%)	14 (46.7%)	17 (63.0%)	0.289
MMF n (%)	7 (23.3%)	5 (18.5%)	0.754
AZA n (%)	1 (3.3%)	1 (3.7%)	1.000
CTX n (%)	1 (3.3%)	0 (0.0%)	1.000

Note: CADM, clinically amyopathic dermatomyositis; ILD, interstitial lung disease; ANA, anti-nuclear antibody; KL-6, krebs von den lungen-6; MDA-5 antibody, anti-melanoma differentiation-associated gene 5 antibody; CK, creatine kinase; HRCT, high-resolution computed tomography; CsA, cyclosporine A; MMF, mycophenolate mofetil; AZA, azathioprine; CTX, cyclophosphamide.

^§^Number of tested cases: pirfenidone add-on group 26 cases, control group 7 cases.
